# Molecular Structure of Photosynthetic Microbial Biofuels for Improved Engine Combustion and Emissions Characteristics

**DOI:** 10.3389/fbioe.2015.00049

**Published:** 2015-04-20

**Authors:** Paul Hellier, Saul Purton, Nicos Ladommatos

**Affiliations:** ^1^Department of Mechanical Engineering, University College London, London, UK; ^2^Institute of Structural and Molecular Biology, University College London, London, UK

**Keywords:** biofuels, combustion, designer fuels, diesel, spark ignition, synthetic biology

## Abstract

The metabolic engineering of photosynthetic microbes for production of novel hydrocarbons presents an opportunity for development of advanced designer biofuels. These can be significantly more sustainable, throughout the production-to-consumption lifecycle, than the fossil fuels and crop-based biofuels they might replace. Current biofuels, such as bioethanol and fatty acid methyl esters, have been developed primarily as drop-in replacements for existing fossil fuels, based on their physical properties and autoignition characteristics under specific combustion regimes. However, advances in the genetic engineering of microalgae and cyanobacteria, and the application of synthetic biology approaches offer the potential of designer strains capable of producing hydrocarbons and oxygenates with specific molecular structures. Furthermore, these fuel molecules can be designed for higher efficiency of energy release and lower exhaust emissions during combustion. This paper presents a review of potential fuel molecules from photosynthetic microbes and the performance of these possible fuels in modern internal combustion engines, highlighting which modifications to the molecular structure of such fuels may enhance their suitability for specific combustion regimes.

## Introduction

The acceptance that the emission of carbon dioxide from the combustion of fossil fuels is contributing to global climate change (Bernstein et al., 2007; Zecca and Chiari, 2010) has driven the recent development of sustainable alternative fuels for internal combustion engines. However, there is now growing concern that production and use of some of the most commonly considered biofuels (bioethanol from fermentation of crop sugars and fatty acid esters from vegetable oils), which compete with food production, may actually offer much smaller reductions in net emissions of fossil CO_2_, relative to the use of fossil fuels, when considering the entirety of the fuel production-to-consumption lifecycle (Overmars et al., 2011).

While there is considerable debate as to the inclusion of factors such as indirect land use changes (ILUC) when undertaking life cycle analysis of biofuels (Ahlgren et al., 2008; Finkbeiner, 2014), such considerations are now being incorporated into legislation. The 2009 European Parliament Renewable Energy Directive stipulates that biofuels must comprise a mandatory 10% minimum of gasoline and diesel consumption for road transport in all EU member states by 2020 (European Commission and European Parliament and Council, 2009). Furthermore, these biofuels must comply with strict sustainability criteria that consider lifecycle savings in greenhouse gas emissions (GHG) relative to fossil fuel equivalents. The proposed strengthening of these criteria to account for ILUC would see the level of renewable fuels from food crops (sugar and starch rich cereals, vegetable oils) capped at 5% of road transport fuels (European Commission, 2012). The remaining 5% must therefore be sourced from advanced biofuels with a low ILUC, such as photosynthetic micro-organisms, lignocellulosic biomass, and various waste streams.

The use of photosynthetic micro-organisms for the production of biofuels is an attractive prospect since, like plants, they harness light energy to synthesize organic molecules, but their cultivation does not require agricultural land. Furthermore, unlike heterotrophic micro-organisms (e.g., *Escherichia coli* or yeast), their growth does not require a crop-derived feedstock such as sucrose. Cultivation can also include the remediation of waste streams, such as carbon dioxide rich power station exhaust gases and nutrient containing wastewater effluent (Lizzul et al., 2014). However, perhaps most exciting is the potential for the application of synthetic biology approaches to produce microbial strains capable of synthesizing designer biofuels. Current biofuels are processed so that the fuel physical properties, and fuel autoignition and combustion phasing characteristics (specified by cetane and octane ratings in diesel and spark ignition combustion, respectively) meet strictly defined standards (European Parliament and Council, 1998, 2009; ASTM International, 2008, 2011). Targeted production of hydrocarbons (HC) with specific molecular structure offers the opportunity to include molecular features that may increase the efficiency of energy release during combustion, or reduce the emissions of harmful pollutants, such as particulate matter (PM) or nitrogen oxides (NOx).

This paper presents an overview of the current range of fuel molecules that might feasibly be produced from photosynthetic micro-organisms, and a concise review of the combustion and emissions characteristics of these potential fuels during both diesel and spark ignition combustion. Possible improvements in the efficiency of fuel energy release or the reduction of exhaust pollutant emissions through alterations of the fuel molecular structures are discussed, in the context of both previous combustion studies and reports of hydrocarbon and oxygenate molecules successfully synthesized in engineered photosynthetic micro-organisms.

## Potential Biofuel Molecules and the Effect of Molecular Structure on Combustion and Emissions

The following sections describe potential fuels for both modern spark ignition engines and modern diesel engines. Fuels for diesel and spark ignition engines are necessarily different in composition due to the combustion strategies employed. In diesel (or compression ignition) engines, following delivery of fuel to the engine combustion chamber, ignition and combustion commences when, due to the high pressure and temperature environment within the combustion chamber, breakdown of fuel molecules and reaction with oxygen in the air results in fuel autoignition. In spark ignition engines, fuel ignition and combustion does not commence until an external ignition source, in the form of a spark, is provided. Fuels which autoignite prior to the provision of the spark produce combustion events, which are uncontrolled, occurring earlier in the engine cycle than intended, and are referred to as engine knock. Therefore, in spark ignition engines, fuels which autoignite (as is required in diesel engines) are undesirable as the engine knock, which such fuels produce, can result in engine damage and higher emissions of regulated pollutants, such as NOx or PM.

### Alkanes and alkenes

**Figure 1:**
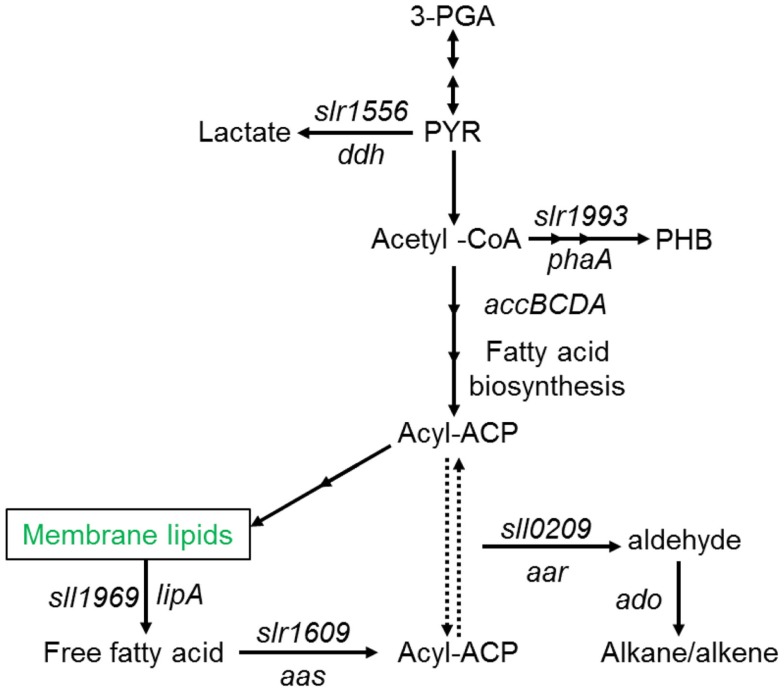
**Schematic overview of fatty acid, alkane (alkene), and main competing metabolic pathways in *Synechocystis* sp. PCC6803**. Redrawn with permission from Wang et al. (2013).

A number of species of cyanobacteria have been seen to naturally produce low amounts of both alkanes and alkenes, with heptadecane and pentadecane observed to be the most abundantly produced in a study of 10 cyanobacterial strains (Schirmer et al., 2010). The metabolic pathway responsible for alkane synthesis via aldehyde intermediates in cyanobacteria, and shown in Figure 1, was first identified by Schirmer et al. (2010). Subsequently, this pathway has been exploited by Wang et al. (2013) for the enhanced production of alkanes and alkenes from *Synechocystis* sp. PCC 6803, with a reported yield of 1.3% of dry cell weight, and the alkane and alkene mixture consisting of primarily heptadecane, heptadecene, and pentadecane. The synthesis of short chain alkenes in cyanobacteria is also possible, with two groups reporting the modification of *Synechocystis* sp. PCC 6803 for production of ethylene (Guerrero et al., 2012; Ungerer et al., 2012). More recently, Kallio et al. (2014) have reported the engineering of *E. coli* to produce propane by introduction a synthetic metabolic pathway that is dependent on fatty acid biosynthesis. As the authors discuss in their paper, it should be possible to introduce such a pathway into a phototrophic organism such as a cyanobacterium. Table 1 shows the molecular structure of these potential alkanes and alkenes from photosynthetic micro-organisms, and also those of other potential fuels discussed in later sections.

**Table 1:** **Molecular structure of selected potential fuels from photosynthetic micro-organisms**.

Alkanes and alkenes	
*n*-heptadecane	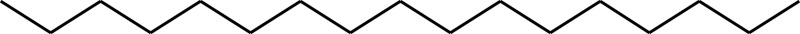
1-heptadecene	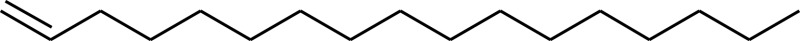
*n*-pentadecane	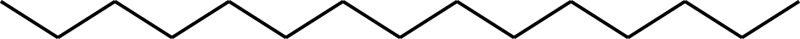
Ethylene	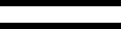
*n*-propane	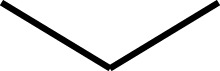

**Alcohols**	

Ethanol	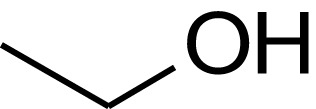
*n*-butanol	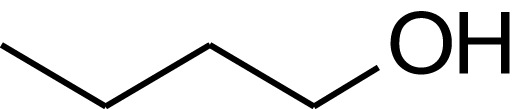
*Iso*-butanol	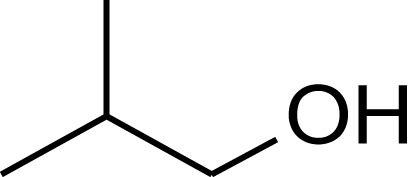

**Fatty acids**	

Palmitic acid (C16)	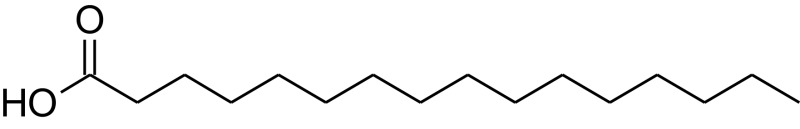

**Terpenes**	

Citronellol	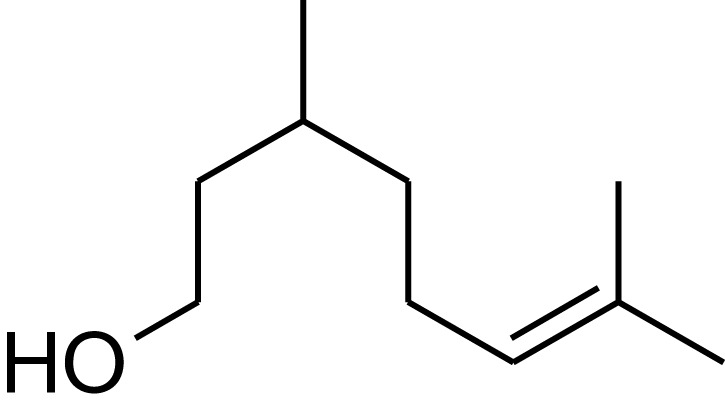
3,7-Dimethyl-1-octanol	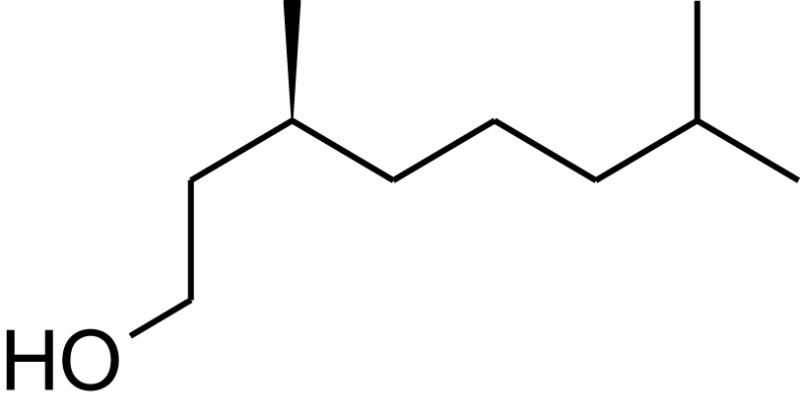
β-caryophyllene	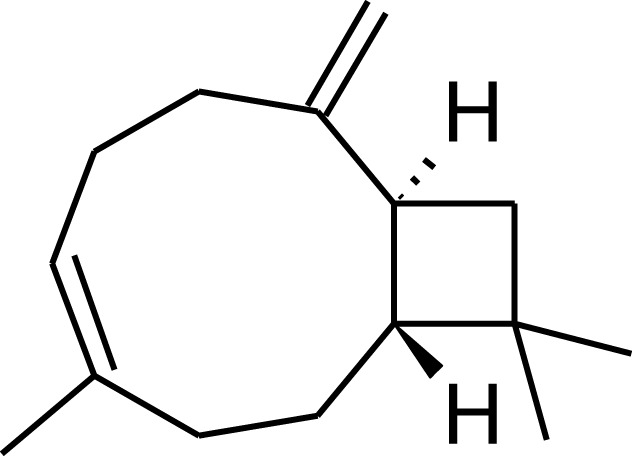
α-bisabolene	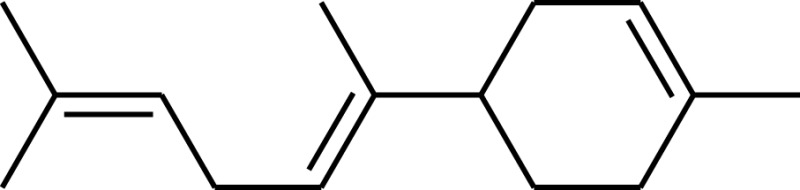

Straight chain and branched (*iso*) alkanes and alkenes are constituent components of both fossil-derived diesel and gasoline fuels. In the case of fossil diesel fuels, alkanes and alkenes from C7 to C16 are typical, with *n*-hexadecane (C16) providing the upper reference point in the cetane number scale. As such, alkanes and alkenes have frequently been utilized as surrogate fuels in place of the complex hydrocarbon mixtures that make up fossil diesel, and the effects of the alkyl chain length and saturation are relatively well understood in the context of diesel combustion characteristics.

**Figure 2:**
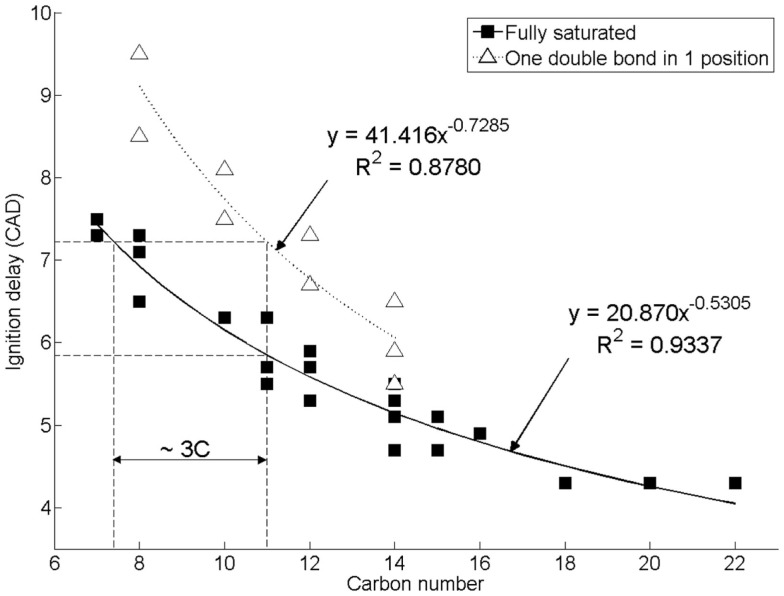
**Effect of increasing alkyl chain length and introducing a double bond on diesel combustion ignition delay at constant fuel injection timing**. Reprinted with permission from SAE paper 2011-01-1922 (Hellier et al., 2011), Copyright ©2011 SAE International.

Figure 2 shows the effect of increasing *n*-alkane straight carbon chain length, and of introducing a double carbon to carbon bond in the 1 position, on the duration of diesel combustion ignition delay when tested as single component fuels at constant engine operating conditions of 1200 rpm, 450 bar injection pressure, constant injection timing of 7.5 CAD before top-dead-center (CAD BTDC), and at a constant engine work output of 4 bar indicated mean effective pressure (IMEP) (Hellier et al., 2011). In direct injection diesel combustion, fuel ignition delay is the duration between the start of fuel injection and fuel autoignition, as signified by the appearance of positive heat release, which indicates that combustion has commenced and is releasing useful energy. It can be seen in Figure 2 that increasing the straight carbon chain length in either an *n*-alkane or 1-alkene reduces the duration of ignition delay, while the presence of a double bond in a 1-alkene increases the duration of ignition relative to the *n*-alkane of equivalent carbon chain length by approximately the same duration as a decrease in *n*-alkane carbon chain length of three carbon atoms. The addition of methyl branches to an alkyl chain to form *iso*-alkanes has also been observed to result in an increase in ignition delay, with the removal of 20% of carbons from the straight chain length and reattached as methyl branches to the straight carbon chain resulting in an increase in the duration of ignition delay of approximately 1.3 CAD (Hellier et al., 2011). Reducing straight alkyl chain lengths, reducing the degree of saturation, or introducing alkyl chain branching are all known to increase the difficulty with which individual hydrogen atoms can be abstracted following fuel injection (Westbrook, 2000). Such hydrogen abstractions are the initiating steps in the low temperature reactions that occur during the ignition delay period, and result in an escalation of temperatures and of the pool of combustion radicals available to the point at which autoignition can occur (Westbrook, 2000).

In the same study of single component *n*-alkanes and 1-alkenes, a strong relationship was observed between the duration of fuel ignition delay and the levels of exhaust NOx emitted (Hellier et al., 2011). Levels of NOx emitted in diesel combustion are known to be strongly dependent on in-cylinder temperatures, as the dominant mechanism of NOx formation is the thermal oxidation of nitrogen [also known as the Zeldovich mechanism (Zeldovich et al., 1947)]. Therefore, the increase in NOx emissions with increasing duration of fuel ignition delay was attributed to the increased time available for fuel and air mixing prior to the start of combustion, which resulted in a larger fraction of premixed combustion and higher peak heat release rates, and thus higher in-cylinder temperatures (Hellier et al., 2011). The same observation has been made in several other studies where diesel fuels of increased cetane number (equivalent to a shorter duration of ignition delay) emitted reduced levels of NOx relative to fuels of lower cetane number (Heywood, 1988; Graboski and McCormick, 1998). A secondary influence of alkyl chain double bonds on the emissions of NOx is through the increase of adiabatic flame temperature, which has been observed to coincide with an increase in NOx emissions, relative to the fully saturated alkyl chain of equivalent length where ignition delay has been equalized through the use of ignition improving additives (Schönborn et al., 2009; Hellier et al., 2013b).

In the context of the C15 and C17 alkanes and alkenes found to be the primary components of cyanobacterial alkane and alkene production, it is interesting to note that under the same experimental conditions as utilized in the engine tests presented in Figure 2, a reference fossil diesel, compliant with current EU specification, exhibited a duration of ignition delay of 7.4 CAD (Hellier et al., 2011). Both *n*-pentadecane and *n*-heptadecane could be expected to exhibit an ignition delay of approximately 5 CAD under the same conditions, while 1-pentadecene might be expected to display an ignition delay of 5.5–CAD (Figure 2). Therefore, it is suggested that the use of C15 and C17 alkanes and alkenes as drop-in blends with current fossil diesels may be limited by the tolerance of the engine control systems for fuels of shorter ignition delays, and thus slightly shorter chain length alkanes and alkenes may provide a more easily integrated alternative.

Fuels that autoignite easily under high temperature and pressure conditions are undesirable in spark ignition combustion as this results in fuel knocking, which are uncontrolled combustion events that result in physical damage to spark ignition engines (Heywood, 1988). Therefore, features of fuel molecular structure that produce a longer duration of diesel ignition delay will generally result in a fuel more suited to spark ignition combustion, as indicated by the octane number of that fuel. In the measurement of fuel octane rating, 2,2,4-trimethylpentane (*iso*-octane) is utilized as a primary reference fuel of octane rating 100 (ASTM International, 2014), indicating a high level of resistance to autoignition. Reports of spark ignition engine testing of liquid alkanes and alkenes other than those used for primary reference fuels (*iso*-octane and *n*-heptane) are very limited, but it can be seen that alkanes and alkenes of significantly shorter carbon chain length than C15 and C17 are required. For example, ethylene has been reported to possess an octane number comparable to commercially available gasoline fuels (Livingston, 1951). Furthermore, propane, in mixtures with other C3 and C4 alkanes and alkenes referred to as LPG, is widely used as a fuel for spark ignition engines and with propane generally exhibiting the greatest level of knock resistance of the mixture components (Morganti et al., 2013).

### Alcohols

**Figure 3:**
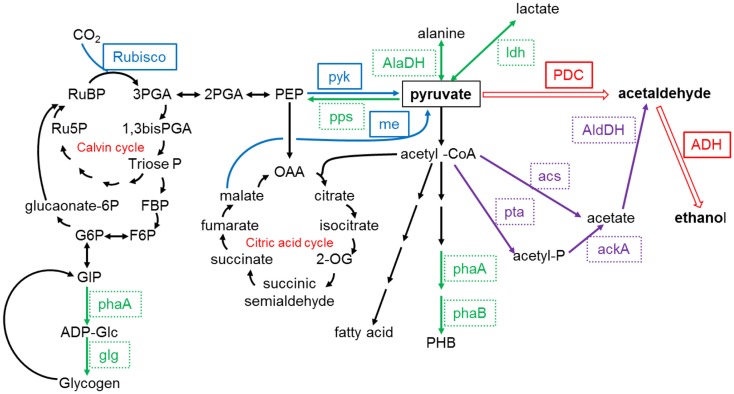
**Pyruvate relevant metabolic pathways in *Synechocystis* sp. PCC6803**. ackA, acetate kinase; acs, acetyl-coenzyme A synthetase; AlaDH, alanine dehydrogenase; ADH, alcohol dehydrogenase; agp, ADP-glucose pyrophosphorylase; glg, glycogen synthase; AldDH, acetaldehyde dehydrogenase; me, malic enzyme; PDC, pyruvate decarboxylase; pps, phosphoenolpyruvate synthase; ldh, lactate dehydrogenase; phaA, PHA-specific β-ketothiolase; phaB, PHA-specific acetoacetyl-CoA reductase; pta, phosphotransacetylase; pyk, pyruvate kinase; Rubisco, ribulose-1,5-bisphosphate carboxylase/oxygenase. Redrawn from Gao et al. (2012) with permission from The Royal Society of Chemistry.

The direct production of ethanol through light-driven CO_2_ fixation by cyanobacteria (as opposed to the traditional method of fermentation of sugars by heterotrophic microbes) has been reported by several researchers (Deng and Coleman, 1999; Dexter and Fu, 2009; Gao et al., 2012). The strategy involves the introduction of genes for pyruvate decarboxylase and alcohol dehydrogenase to convert pyruvate to ethanol, as shown in Figure 3. Deng and Coleman (1999) introduced gene sequences from the bacterium *Zymomonas mobilis* into *Synechococcus elongatus*, which resulted in ethanol production and its subsequent diffusion from the cells to the culture medium. More recently, both Dexter and Fu (2009) and Gao et al. (2012) integrated sequences from *Zymomonas mobilis* into *Synechocystis* PCC 6803 for ethanol production via that of acetaldehyde, as shown in Figure 2, with a final ethanol concentration of 5.5 g/L after a cultivation period of 26 days reported by the latter (Gao et al., 2012). Several companies including Alginol in the USA^1^ and Photanol in the Netherlands^2^ are currently commercializing such technology.

**Figure 4:**
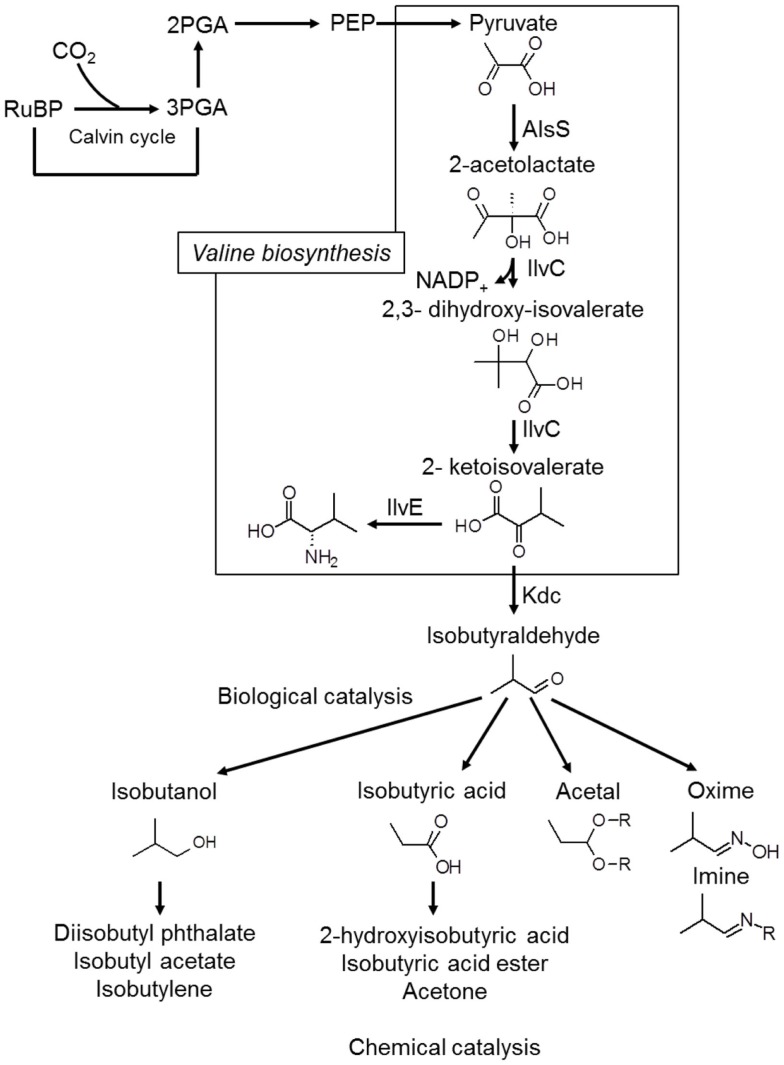
**The Kdc-dependent synthetic pathway for isobutyraldehyde production**. AlsS, acetolactate synthase; IlvC, acetohydroxy acid isomeroreductase; IlvD, dihydroxy-acid dehydratase; Kdc, 2-ketoacid decarboxylase. Redrawn from Atsumi et al. (2009) with permission from the Nature Publishing Group.

*Iso*-butanol production has also been achieved, with Atsumi et al. (2009) reporting production of 450 mg/L of *iso*-butanol over the course of 6 days. This was obtained with a modified strain of *Synechococcus* elongatus PCC7942, which first produces isobutyraldehyde, with the aldehyde subsequently converted to *iso*-butanol (Atsumi et al., 2009), as shown in Figure 4. The same strain was also modified by Oliver et al. (2013) to produce 2,3-butanediol at yields up to 238 mg/L.

Ethanol has a long history of use as a drop-in blend with fossil gasoline for spark ignition combustion, with industrial production of ethanol from the microbial fermentation of biomass (Heywood, 1981; Krouwel et al., 1983; Rosillo-Calle and Walter, 2006; Ezeji et al., 2007). Both ethanol and *n*-butanol have been studied as blends with fossil gasoline in spark ignition engines, with the higher energy density of *n*-butanol relative to ethanol resulting in lower rates of fuel consumption (Wallner et al., 2009). The higher octane rating of ethanol relative to fossil gasoline allows for engines to be operated with a more advanced spark timing (without risking the occurrence of engine knock), which results in higher engine thermal efficiencies and also higher emissions of NOx due to an increased residence time of combustion gases at temperatures sufficient for NOx production (Nakama et al., 2009; Wallner et al., 2009). Spark ignition engine testing of unblended pure *n*-butanol has found that it exhibits combustion characteristics similar to fossil gasoline (Szwaja and Naber, 2010), with higher relative emissions of unburnt HC and carbon monoxide (Gu et al., 2012). Both HC and CO can arise due to incomplete fuel combustion, either attributable to insufficient availability of oxygen or in-cylinder temperatures for complete combustion, with reduced in-cylinder temperatures attributable to the higher latent heat of vaporization of *n*-butanol relative to fossil gasoline plausible and further supported by an observed decrease in emissions of NOx (formation rates of which are temperature dependent) (Gu et al., 2012).

The use of *iso*-butanol in spark ignition engines has received less attention, with Irimescu (2012) reporting a decrease in engine thermal efficiency due to incomplete evaporation of *iso*-butanol following port injection. However, shock tube measurements of the ignition delay of butanol isomers have found the ignition delay of *iso*-butanol to be longer than *n*-butanol, suggesting *iso*-butanol to possess a knock resistance between that of *n*-butanol and ethanol. Therefore, given the potential for *iso*-butanol production from cyanobacteria, it is tentatively suggested that in the context of spark ignition combustion, *iso*-butanol might combine the higher energy density of *n*-butanol with an improved knock resistance that would result in higher engine thermal efficiencies.

Due to their low cetane number (Yanowitz et al., 2014), short chained alcohols such as ethanol, *iso*-butanol, and *n*-butanol have not been widely considered as single component fuels for diesel engines. However, both ethanol and *n*-butanol have been utilized in engine studies as blends with either fossil diesel fuel or biodiesel (Lapuerta et al., 2009; Sayin, 2010; Rakopoulos et al., 2011; Ballesteros et al., 2012; Giakoumis et al., 2013). Addition of either ethanol or *n*-butanol at levels up to 30% has been observed to result in an increased duration of fuel ignition delay and a reduction in the emission of PM (Giakoumis et al., 2013; Kumar et al., 2013). The production of PM during diesel combustion occurs in fuel rich zones of the combustion chamber (Tree and Svensson, 2007), and so an increase in fuel ignition delay, which allows more time for fuel and air mixing prior to the start of combustion, will likely result in fewer fuel rich zones and reduced particulate formation. The blending of *iso*-butanol with fossil diesel at levels up to 20% has been observed to result in a decrease in emissions of CO and NOx, with a significant increase in HC emissions (Karabektas and Hosoz, 2009; Pal et al., 2013). At a blend level of 40%, a decrease in exhaust temperature and engine thermal efficiency has been observed and attributed to the higher heat of vaporization of *iso*-butanol relative to fossil diesel (Al-Hasan and Al-Momany, 2008).

### Fatty acids and other acids

Many species of eukaryotic microalgae are known to synthesize and accumulate triglycerides of saturated and unsaturated chain lengths of between 16 and 20 carbon atoms (Griffiths et al., 2012; Jones et al., 2012), with Lü et al. (2011), reporting accumulation of such neutral lipids at levels up to 60% when considered on a dry cell weight basis. Cyanobacteria do not show such accumulation of triglycerides; however, *Synechococcus* sp. PCC 7002 was engineered by Ruffing (2014) to produce free fatty acids at yields of greater than 130 mg/L, although no specification of the free fatty acids produced was provided. Liu et al. (2011) modified *Synechocystis* sp. PCC 6803 to produce and secrete free fatty acids in the range of C10–C18, with saturated C16 fatty acids the most abundant at levels as high as 60% wt/wt. The synthesis of other acids is also possible, with Varman et al. (2013) modifying *Synechocystis* sp. PCC 6803 to produce d-lactic acid.

The use of long alkyl chain fatty acids in the form of triglycerides from vegetable sources, such as sunflower, rapeseed and soy, received significant attention in the early 1980s as an alternative fuel for diesel combustion (Cruz et al., 1981; Pryde, 1983). However, the use of triglycerides was quickly found to adversely affect the durability and performance of diesel engines (Pryde, 1983; Ryan et al., 1984); many of these issues were found to be diminished in severity through trans-esterification of the triglycerides using short chain alcohols to form esters (Harrington, 1986; Graboski and McCormick, 1998). Such fatty acid esters are now commonly referred to as biodiesel, and are mandatorily present in European consumer diesel at levels up to 10% v/v (European Commission and European Parliament and Council, 2009).

Many of the subsequent studies of fatty acid esters have focused on the performance of these biodiesels from various sources relative to existing fossil diesel fuels, and several extensive reviews have already been presented on the subject (Graboski and McCormick, 1998; Szybist et al., 2007; Basha et al., 2008; Lapuerta et al., 2008; Atadashi et al., 2010; Hoekman and Robbins, 2012). However, in the context of the production of fatty acids from photosynthetic micro-organisms (Table 1), where there may be the potential for a degree of selectivity over the molecular structure of the fatty acids produced, it is more interesting to focus on how the alkyl chain length and degree of saturation present in fatty acids (and the subsequently produced fatty acid esters, which will preserve the initial alkyl chain structure) impacts on combustion and emissions.

**Figure 5:**
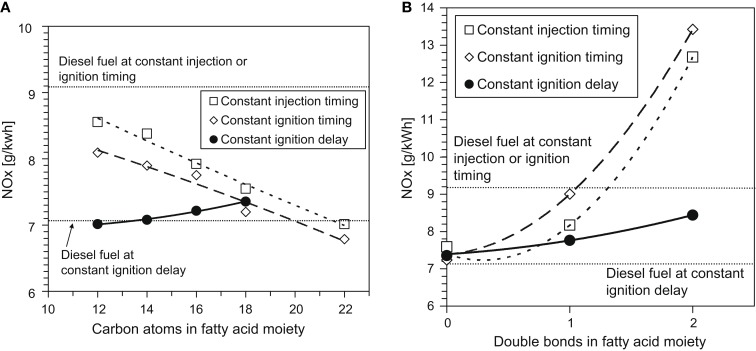
**Effect of C18 fatty acid methyl ester (A) alkyl chain length and (B) number of double bonds in the alkyl chain on NOx emissions**. Adapted from Schönborn et al. (2009) with permission from Elsevier.

Figure 5 shows the effect of increasing the fatty acid methyl ester (FAME) saturated alkyl chain length and increasing the number of double bonds within a constant alkyl chain length on NOx emissions in diesel engine tests of single component fuels (Schönborn et al., 2009). The tests were conducted at constant engine operating conditions, two fuel injection timings at which the duration of ignition delay varied according to the fuel (constant injection and constant ignition), and a third injection timing where an ignition improving additive was utilized to equalize the duration of ignition delay for all fuels. It can be seen that increasing the fatty acid alkyl chain length (Figure 5A) decreases NOx emissions, while increasing the number of double bonds present in the fatty acid alkyl chain (Figure 5B) increases NOx emissions. With increasing fatty acid alkyl chain length, a reduction in the duration of ignition delay was observed, while increasing the number of double bonds present extended the duration of ignition delay (Schönborn et al., 2009). These observations are in agreement with other studies (Allen et al., 2013; Pinzi et al., 2013), and suggest that the primary influence of the fatty acid alkyl chain molecular structure on NOx emissions is through the duration of ignition delay and subsequent in-cylinder thermal conditions, as outlined in the discussion of alkane and alkene combustion (see Alkanes and Alkenes). However, in Figures 5A,B, it is apparent that at constant ignition delay timing, NOx emissions increase with both fatty acid alkyl chain length and the number of double bonds present. This is suggestive of the secondary influence of alkyl chain structure via the adiabatic flame temperature, as was highlighted in Section “Alkanes and Alkenes.”

**Figure 6:**
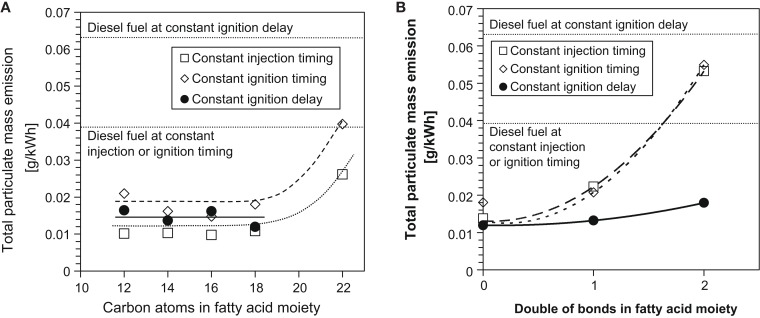
**Effect of C18 fatty acid methyl ester (A) alkyl chain length and (B) number of double bonds in the alkyl chain on total particulate mass emissions**. Adapted from Schönborn et al. (2009) with permission from Elsevier.

Figure 6 shows the effect of increasing the FAME saturated alkyl chain length and increasing the number of double bonds within a constant alkyl chain length on total particulate mass emissions in diesel engine tests of single component fuels (Schönborn et al., 2009). At all injection timings, it can be seen that between fatty acid alkyl chain lengths of 12 and 18, there is no apparent impact on emissions of PM, with only the C22 fatty acid ester emitting higher levels of PM (Figure 6A). This may possibly be attributable to the reduced oxygen content and higher viscosity of the C22 ester, both of which might be expected to impede fuel air mixing and favor soot formation (Pinzi et al., 2013). In Figure 6B, it can be seen that increasing the number of double bonds within the fatty acid ester alkyl chain increases the level of PM at all injection timings. The presence of double bonds within fatty acid ester alkyl chains has been suggested as increasing the formation of soot precursors and thus total levels of PM emitted (Schönborn et al., 2009). A decreasing degree of fatty acid ester alkyl chain saturation has also been linked to a decrease in oxidative stability, and the products of FAME oxidation (primarily acids and other short to long chain oxygenated compounds) can negatively impact on engine performance and durability in a manner similar to triglycerides (Knothe, 2006; Bannister et al., 2011).

At present, the vast majority of fatty acid esters produced as diesel fuels are the result of trans-esterification of triglycerides with methanol. However, it is possible to undertake trans-esterification and produce fatty acid esters with alcohols other than methanol, and this presents an additional opportunity for utilization of short chain alcohols from photosynthetic micro-organisms (see Alcohols) in diesel combustion.

**Figure 7:**
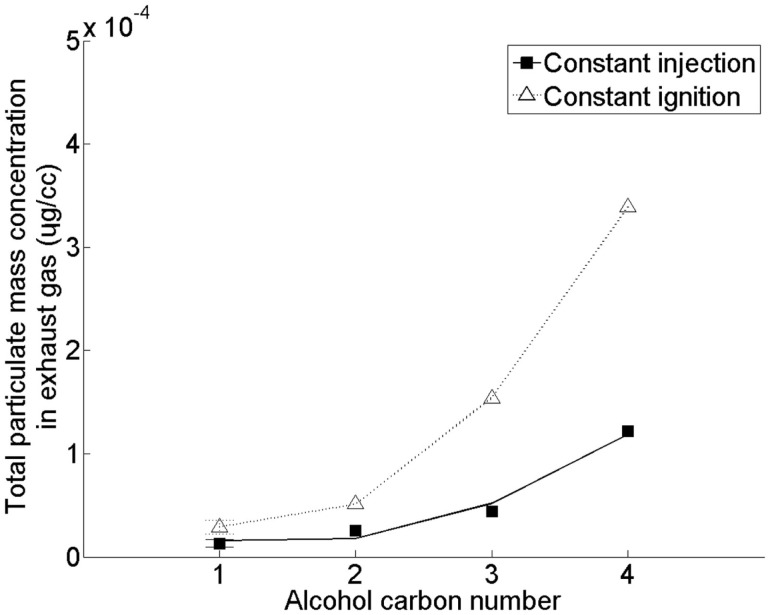
**Effect of C18 fatty acid saturated ester alcohol moiety straight carbon chain length on total particulate mass emissions**. Adapted with permission from Hellier et al. (2012). Copyright ©2012 American Chemical Society.

Figure 7 shows the effect of increasing the straight carbon chain length of the alcohol moiety of a saturated fatty acid ester (of alkyl moiety chain length 18) on emission of PM (Hellier et al., 2012). In this study, no clear influence of substituting the methyl alcohol moiety of the fatty acid ester with ethyl, propyl, and butyl alcohol moieties on the duration of fuel ignition delay was observed (Hellier et al., 2012) [in agreement with other investigations (Allen et al., 2013)]. However, in Figure 7 it can be seen that an increase in the fatty acid ester alcohol moiety results in a significant increase in the production of PM. This increase with alcohol moiety length coincides with a decreasing molecular oxygen content and increasing viscosities and boiling points (Hellier et al., 2012), all of which can be expected to result in increased particulate formation and emission (Tree and Svensson, 2007). A saturated C18 fatty acid *iso*-butyl ester was also tested under the same conditions as the results presented in Figure 7, and found to emit levels of PM similar to those observed from the equivalent *n*-propyl ester (Hellier et al., 2012). Therefore, in the utilization of short chain alcohols from photosynthetic micro-organisms to form fatty acid esters, consideration should be given as to whether the resultant physical properties of the ester may produce elevated emissions of PM.

The high cetane number (Yanowitz et al., 2014) (and thus likely low octane number) of long chained fatty acid esters has precluded consideration of the use of such esters (or fatty acids) in spark ignition combustion. However, shorter fatty acid esters such as methyl butanoate and ethyl levulinate have been reported as possessing very low cetane numbers (Yanowitz et al., 2014) and thus propensity to autoignite, and so, dependent on the physical properties of such esters, may have potential as spark ignition fuels.

### Terpenes

**Figure 8:**
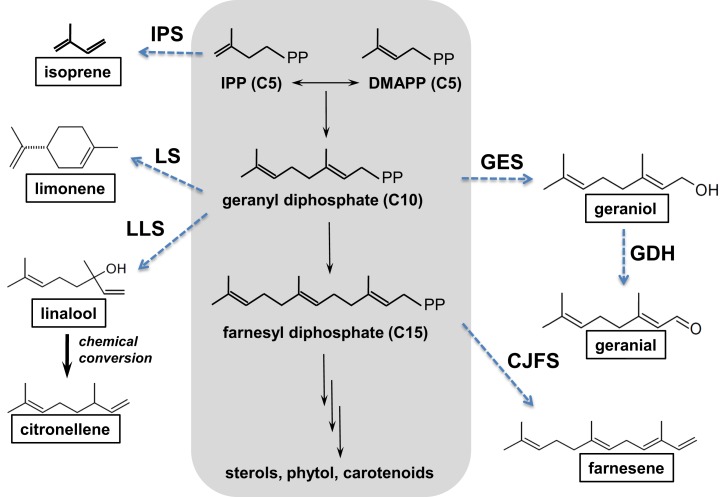
**The isoprenoid pathway (highlighted in gray) and examples of possible pathways (dashed arrows) that could be introduced to produce novel hydrocarbons (boxed)**. CJFS, (*E*)-β-farnesene synthase; IPS, isoprene synthase; GDH, geraniol dehydrogenase; GES, geraniol synthase; LLS, linalool synthase; LS, limonene synthase. Reproduced with permission from Hellier et al. (2013a).

The potential of cyanobacteria for the production of terpenes has recently attracted interest (Hellier et al., 2013a), with the possible addition of novel plant enzymes to the terpenoid (or isoprenoid) pathway, as illustrated in Figure 8, resulting in a wide range of potential products based on the C5 isoprene unit. Addition of the individual isoprene units allows construction of larger molecules (C10, C15), while inclusion and manipulation of oxygenated functional groups is also possible. To date, the synthesis of both isoprene (Lindberg et al., 2010) and β-caryophyllene (Reinsvold et al., 2011) has been demonstrated in *Synechocystis* sp. PCC 6803, while Davies et al. (2014) engineered *Synechococcus* sp. PCC 7002 to produce 4 and 0.6 mg/L of limonene and α-bisabolene, respectively.

While there is a great deal of potential for producing fuels of varying molecular structure via the isoprenoid pathway described in Figure 8, isoprenoids and terpenes have received little attention in the context of either diesel or spark ignition combustion. In a recent study, the present authors investigated the combustion and emissions characteristics of a range of terpenes, based on the molecules achievable through modification of the isoprenoid pathway, as shown in Figure 8 (Hellier et al., 2013a).

**Figure 9:**
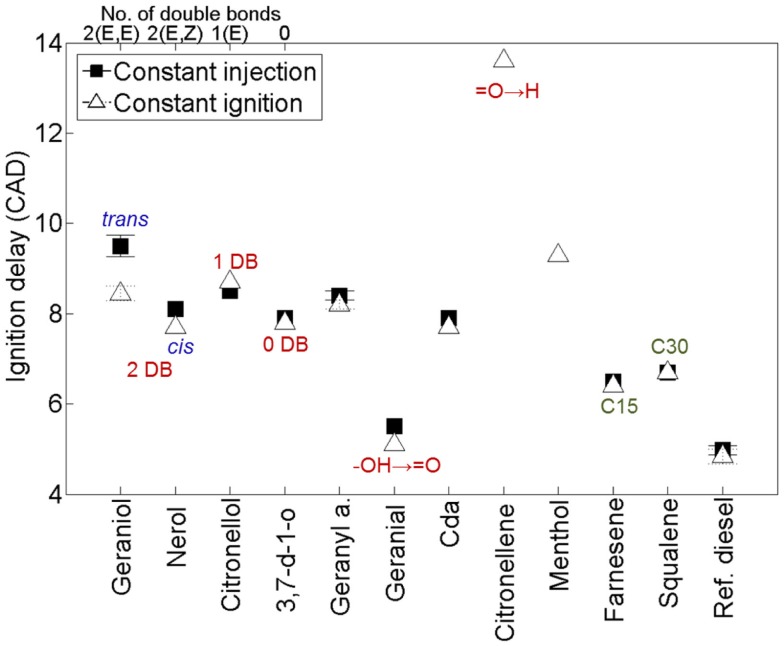
**Effect of varying terpene molecular structure on the duration of ignition delay**. Adapted from Hellier et al. (2013a).

Figure 9 shows the ignition delays of the 11 terpenes compared to a reference fossil diesel when tested as single component fuels in a modern diesel engine (Hellier et al., 2013a). It can be seen that changes to the molecular structure of the terpenes results in a significant variation in the duration of the ignition delay, with some fuels exhibiting a duration of ignition delay close to that of the reference fossil diesel. In agreement with observations made of alkenes and fatty acid esters (see Alkanes and Alkenes and Fatty Acids and Other Acids), decreasing the number of double bonds reduces the duration of ignition delay (Figure 9); i.e., from 2 to 1 to 0 alkyl chain double bonds in geraniol, citronellol, and 3,7 dimethyl-1-octanol, respectively. The presence of an oxygenated functional group is beneficial to the fuel reactivity at these conditions, with the alkene citronellene displaying a much longer duration of ignition delay than geraniol, which has an equivalent alkyl chain structure but includes an alcohol group. Combination of the isoprene unit to form farnesene (C15) and squalene (C30) reduces the duration of ignition delay relative to geraniol, despite the absence of an oxygenated functional group (Figure 9). Perhaps, most striking is the extent of the reduction in ignition delay with removal of a single hydrogen atom from the alcohol group of geraniol to form geranial (Figure 9). This is likely attributable to the requirement for alcohols to be converted to an intermediate aldehyde during the low temperature reactions that occur during the ignition delay period (Salooja, 1965).

**Figure 10:**
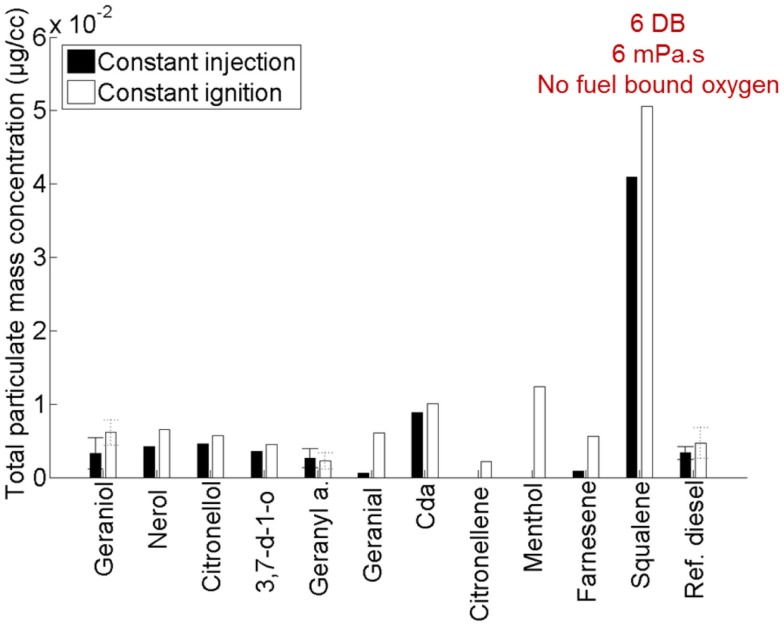
**Effect of varying terpene molecular structure on the total particulate mass emitted**. Adapted with permission from Hellier et al. (2013a).

Figure 10 shows the total particulate mass emitted by the 11 terpenes relative to a reference fossil diesel when tested as single component fuels in a modern diesel engine (Hellier et al., 2013a). Immediately apparent are the significantly higher emissions of PM from squalene relative to the other terpenes considered. Squalene was found to be the most viscous of all of the terpenes (Hellier et al., 2013a), and it is likely that this high viscosity (which would have impeded fuel and air mixing), in addition to the high number of double bonds present and lack of fuel bound oxygen, results in the higher levels of PM emission observed (Figure 10).

In the same study, the present authors also considered the potential of two of the terpenes which had been found to exhibit the longest duration of diesel combustion ignition delay, citronellene and linalool (which did not combust and is thus not present in Figure 9), in the context of spark ignition combustion (Hellier et al., 2013a). Linalool and citronellene were blended with fossil gasoline at levels up to 65 and 50% wt/wt, respectively, and were both found to combust in a steady manner in a single cylinder spark ignition engine.

## Conclusion and Future Challenges

Genetic engineering of photosynthetic micro-organisms presents the opportunity to produce a wide range of fuel molecules for diesel and spark ignition combustion directly from CO_2_. It is clear that relatively minor modifications to the molecular structure of these potential fuels can have significant impact with regards to the efficiency of energy release and emissions of pollutants during combustion, and considering the various biofuel molecules currently achievable from engineered micro-organisms, the following conclusions are drawn:

1. C15 and C17 alkanes and alkenes are suitable for diesel combustion, and while the shorter ignition delay of these molecules relative to fossil diesel might result in lower NOx emissions, it could also limit the use of such long chain molecules as drop-in alternatives. For spark ignition combustion, alkanes and alkenes that do not autoignite easily are required to prevent engine knocking, and this could be achieved through shorter, less saturated, and more branched alkyl chains. For example, *iso*-octane is a suitable fuel for spark ignition combustion, while *n*-octane exhibits similar ignition characteristics as diesel fuels from fossil sources. Therefore, modification of metabolic pathways to control the degree of branching in produced alkanes and alkenes is a potential means by which fuels specific to spark ignition or diesel combustion may be targeted.
2. Short chain alcohols such as ethanol, *n*-butanol, and *iso*-butanol are suitable fuels for spark ignition combustion. Increasing the alcohol carbon chain length from ethanol to *n*-butanol reduces engine thermal efficiency through reduced knock resistance, but also reduces fuel consumption due to the increased energy density of *n*-butanol relative to ethanol. The presence of alkyl chain branching in *iso*-butanol is likely to increase knock resistance relative to *n*-butanol, while preserving the higher energy density of *n*-butanol. In diesel combustion, the blending of short chain alcohols as minor components with fossil diesel results in a significant increase in the duration of ignition delay, and alcohols of alkyl chain lengths longer than that of *n*-butanol (and therefore shorter durations of ignition delay) are required for use at higher levels.
3. Long chain fatty acids (C16–C20) can be trans-esterified with short chain alcohols to form fatty acid esters that are well suited to diesel combustion. Decreasing fatty acid ester alkyl chain lengths or the degree of saturation increases the duration of diesel ignition delay, leading to an increase in NOx emissions, while increasing the alkyl chain length or degree of saturation can increase emissions of PM. Trans-esterification of fatty acids with short chain alcohols other than methanol presents a route for utilization of ethanol and butanol from photosynthetic micro-organisms in diesel combustion, but an increase in physical properties such as boiling point and viscosity with increasing carbon chain length of fatty acid ester alcohol moieties can increase emissions of PM. The use of long chain fatty acids for conventional spark ignition combustion is not feasible due to high boiling point and likely very low knock resistance. In addition, the need for a further processing step (trans-esterification to fatty acid esters for diesel combustion) reduces the positive impacts of production from photosynthetic micro-organisms.
4. Modification of the isoprenoid pathway permits the synthesis of a range of molecules suitable for diesel and spark ignition combustion. The combination of multiple isoprene units (C5) to form larger molecules (C15–C30) reduces diesel combustion ignition delay, but can also increase emissions of PM. Inclusion of an oxygenated functional group in a C10 terpene can also reduce diesel combustion ignition delay, with minor modifications to the functional group (OH → H) resulting in further significant reductions in ignition delay. The absence of an oxygenated functional group in a C10 terpene, or movement of an oxygenated functional group toward the center of the molecule can result in a fuel suitable for spark ignition combustion. This suggests that production may be tuned to either fuels for spark ignition or diesel combustion through relatively minor modifications of the isoprenoid pathway.

There are, of course, major hurdles to translating this promising technology into a viable commercial industry, not least the major biological challenges associated with creating engineered strains capable of producing fuel molecules at significant levels within a large-scale industrial process (Nozzi et al., 2013). While the introduction and expression of foreign genes in cyanobacteria and microalgae is routine for a handful of species (Lü et al., 2011; Berla et al., 2013), the transfer of such technology to more suitable industrial strains still represents a major hurdle. One example of the challenges faced is that many cyanobacterial species possess restriction enzyme systems that efficiently degrade any foreign DNA introduced into the cell (Singh et al., 2011). Once the genetic engineering technology has been established, the next problem is predicting and controlling the effect of novel biosynthetic enzymes on the native metabolism of the micro-organism, since any new biosynthetic pathway needs to be balanced to minimize bottlenecks and maximize product yield, but without compromising the viability of the cell (e.g., by depleting precursors that are required for essential cellular components during growth). Ensuring such flux balance requires the development of sophisticated metabolic models that allow design of new pathways and prediction of how they will perturb metabolite flux (Berla et al., 2013).

A further challenge is how to overcome the significant cytotoxicity of many of the biofuel molecules. While this issue can be addressed in some cases by selecting fuel molecules that show the lowest toxicity to the chosen cyanobacterial host, such as alkanes rather than their equivalent alcohols (Kämäräinen et al., 2012), an additional approach might involve the creation of strains with greater tolerance to toxic products or intermediates through directed evolution strategies (Lü et al., 2011). Other strategies might involve selecting volatile or membrane soluble products that readily diffuse out of the cell into the medium or head-space, or introducing efflux pumps to mediate product transport out of the cell so that the intracellular concentration is kept below the toxic level (Dunlop et al., 2011). Ultimately, biofuel production might require the complete decoupling of cell viability from biofuel synthesis by engineering into the strain tightly controlled inducible gene systems to switch on synthesis once a cell culture reaches stationary phase (Berla et al., 2013).

Finally, a significant issue is the difficulty of achieving a positive energy balance for an industrial process that uses solar energy to create biomass (Cotton et al., 2015). Light-to-biomass energy conversion by photosynthesis is an inherently inefficient process, with a theoretical maximum conversion rate of 12.6%, and a practical maximum in the field of <3% (Zhu et al., 2010). As a result, the industrial cultivation and downstream processing of photosynthetic micro-organisms may result in a potentially futile process where a higher level of input energy (likely at present to be fossil-derived) is required for production than is actually contained in the hydrocarbon product (Cotton et al., 2015). Addressing this issue would require further strain engineering to improve the overall photosynthetic efficiency, together with the use of sustainable sources of input energy (Cotton et al., 2015). Alongside these many biological challenges are also the daunting engineering, economic and environmental challenges of producing any biofuel from a genetically engineered photosynthetic micro-organism on a massive industrial scale, and at a low enough cost that it can make a meaningful contribution to the provision of fuels to the transport sector.

## Conflict of Interest Statement

The authors declare that the research was conducted in the absence of any commercial or financial relationships that could be construed as a potential conflict of interest.

## Footnotes

^1^http://www.algenol.com

^2^http://www.photanol.com

## Abbreviations

3, 7-d-1-o, 3, 7 dimethyl-1-octanol; BTDC, before top-dead-center; CAD, crank angle degree; CO, carbon monoxide; CO_2_, carbon dioxide; FAME, fatty acid methyl ester; GHG, greenhouse gas emissions; HC, hydrocarbons; ILUC, indirect land use changes; IMEP, indicated mean effective pressure; NOx, nitrogen oxides; PM, particulate matter.
